# Unexpected Sequela of Shoulder Dystocia: Neonatal Humerus Fracture

**DOI:** 10.7759/cureus.107288

**Published:** 2026-04-18

**Authors:** Zaineb Ahmad, Carly Salvesen, Gabrielle Brini, Madhu Purushothaman

**Affiliations:** 1 Pediatrics, Touro College of Osteopathic Medicine, Middletown, USA; 2 Pediatrics, Garnet Medical Center, MIddletown, USA

**Keywords:** mcroberts maneuver, neonatal care, obstetrics emergency, obstetrics & gynecology, shoulder dystocia

## Abstract

Shoulder dystocia is an obstetric emergency defined by failure of the fetal shoulders to deliver after expulsion of the head, most often due to impaction of the anterior shoulder behind the pubic symphysis. Although uncommon and considered largely unpredictable, shoulder dystocia carries the risk of significant neonatal morbidity. While brachial plexus injury is the most frequently reported complication, skeletal injuries such as humeral fractures are less commonly described.

A 28-year-old primigravid woman with obesity and anemia presented at 38 weeks and 6 days of gestation with spontaneous rupture of membranes and underwent spontaneous vaginal delivery. Shoulder dystocia was identified following delivery of the fetal head. Initial management with the McRoberts maneuver and suprapubic pressure was unsuccessful, followed by unsuccessful attempts at the Woods screw maneuver. Delivery of the posterior arm successfully resolved the dystocia. Post-delivery examination revealed swelling and crepitus of the neonate’s right upper extremity with intact perfusion, symmetric Moro reflex, and preserved spontaneous movement. Radiographs confirmed a right mid-diaphyseal humeral fracture with mild angulation and overlap. Conservative management with immobilization and analgesia was initiated. Follow-up imaging at three weeks demonstrated appropriate fracture healing, and the infant remained neurologically intact.

Humeral fracture is a rare but recognized complication of shoulder dystocia, particularly following posterior arm delivery. Although such fractures may raise concern for birth trauma, they are typically associated with excellent outcomes when promptly identified and managed conservatively. This case illustrates appropriate escalation of shoulder dystocia maneuvers in accordance with established protocols and emphasizes the importance of thorough neonatal assessment to distinguish skeletal injury from neurologic compromise. This report highlights neonatal humeral fracture as an uncommon complication of shoulder dystocia and reinforces that, despite the urgency of delivery, careful technique and prompt postnatal evaluation can result in favorable outcomes with minimal long-term morbidity.

## Introduction

Shoulder dystocia is an “unpredictable and unpreventable" obstetrical emergencies that occur during vaginal delivery when traction of the fetal head does not result in further delivery of the shoulder because of impaction anteriorly at the pubic symphysis and posteriorly at the sacral promontory [1]. A classical clinical indication of shoulder dystocia is the “turtle sign,” when the fetal head retracts back into the perineum after previously being delivered. This complication of delivery is rare, occurring in about 1% of nulliparous women with vaginal deliveries, with a higher risk of 7% in women with a prior history of shoulder dystocia who have a subsequent vaginal delivery [2]. Occurrence is driven by a complex interplay of factors, in which maternal and fetal anatomy play a central role. 

Maternal pelvic morphology and fetal macrosomia influence the likelihood of shoulder dystocia. With regards to maternal anatomy, the fetus must pass through the pelvic inlet, followed by lesser pelvis, or commonly known as true pelvis, then lastly the pelvic outlet [3]. The narrowest aspect of this track is the passage through the obstetric conjugate, which is measured from the sacral promontory to pubic symphysis and averages 11.5 to 12 cm for the gynecoid-shaped pelvis [3]. Delivery can be further complicated by the size and proportions of the fetus. Fetal macrosomia is associated with increased risk of shoulder dystocia due to the larger anterior-posterior and transverse diameters of the head and shoulders, respectively. In a study with 13,277 total births, there were 221 cases complicated by shoulder dystocias, 50.7% of the infants were <4000 g [4]. As shown, fetal macrosomia is associated with higher rates of shoulder dystocia, and one study showed that additional increments of 500 g were associated with a 10-fold increased incidence when birthweight was over 4500 g [4,5]. Although shoulder dystocia is an uncommon complication of vaginal delivery, factors of female pelvis shape and fetal size can compound and increase the probability of complications. Clinicians are routinely trained in the management of shoulder dystocia to prevent adverse maternal and fetal outcomes. 

If shoulder dystocia is identified during delivery, basic protocols are initiated, and subsequent maneuvers are performed by the clinician to relieve the shoulder. The promptness of protocol activation lies in the fact that compression of the umbilical cord leaves a previously well-oxygenated infant only five minutes until the risk of asphyxiation injuries increases significantly [6]. The order of protocol generally follows documentation of time, cessation of labor pushing, acquiring more staff if needed, decreasing downward traction of the fetal head, draining the bladder if distended, and identifying the shoulder position during the pause between contractions [6]. Upon completion of such protocol, the current guidelines divide maneuvers into primary, secondary, and tertiary. Primary maneuvers include McRoberts, Rubin, and delivery of the posterior arm in order of invasiveness. In the event of the fetal forearm or hand not being reachable due to location relative to the pelvic brim, two secondary maneuvers include axillary traction of the posterior shoulder and the Woods screw maneuver. These different types of maneuvers are an effort to reduce the severity of asphyxia, force of traction, external downward suprapubic pressure, twisting, and torquing of the fetus.

Despite the implementation of protocols and creation of labor maneuvers for this obstetrical emergency, there can be fetal and maternal complications. In a recent study analyzing fetal complications associated with shoulder dystocia, a promising 95% of babies acquired no complications [6]. Such complications from most prevalent to least prevalent include transient brachial plexus injuries, clavicular fracture, humeral fracture, permanent brachial plexus injury, hypoxic ischemic encephalopathy, and death, with humeral fracture ranging from 0.1 to 4.2 percent [6]. This case report will highlight the management and long-term effects of the less common outcome of a humeral fracture.

## Case presentation

A 28-year-old G1P0 complicated by obesity (BMI 42.34) and anemia presented to the ED at 38 weeks and 6 days with spontaneous rupture of the membrane (SROM). The patient was delivered via spontaneous vaginal delivery with a birth weight of 3,390 g. After the delivery of the newborn head, the anterior shoulder was unable to be palpated, and the mother was placed in McRoberts with suprapubic pressure applied; this was unsuccessful. The Woods Maneuver was then performed left and right, but this was also unsuccessful. The right arm was then palpable across the neonatal right chest, and subsequent delivery of the posterior arm was performed with unquantified traction force applied (Table 1). A right humeral fracture was suspected due to the nature of the delivery. At the time of delivery, the newborn had an Apgar score of 7 at 1 minute, which improved to 9 at 5 minutes. Upon examination by the neonatologist after the delivery, the right arm displayed crepitus and swelling consistent with shoulder dystocia. Moro reflex was assessed and was symmetrical. Pulses were strong, and the limb was pink and warm, indicating adequate perfusion. Normal spontaneous movement was observed in the right arm, with the patient able to abduct at the shoulder, flex at the elbow, extend the wrist, and open the hand. These movements were not indicative of brachial plexus injury.

**Table 1:** Shoulder dystocia event timeline and maneuver sequence All five maneuvers were performed within a 40-second interval. Dystocia was recognized and help was called simultaneously. Physician and additional staff arrived within 90 seconds of recognition. NICU attendance was not required. All maneuvers were performed by the attending provider.

Case Overview	
Shoulder dystocia present	Yes
Anterior shoulder	Right
Gentle traction attempt	Yes, assisted by maternal expulsive forces
Event Timeline	
Time recognized	09/22/2025 10:45:00
Help called	09/22/2025 10:45:00
Physician/Provider arrived	09/22/2025 10:46:30
Additional staff arrived	09/22/2025 10:46:50
NICU arrived	N/A
Maneuver Sequence	
1st maneuver	McRoberts maneuver — 10:45:00
2nd maneuver	Suprapubic pressure — 10:45:10
3rd maneuver	Woods screw maneuver — 10:45:20
4th maneuver	Woods screw maneuver — 10:45:30
5th maneuver	Delivery of posterior arm — 10:45:40

**Figure 1:**
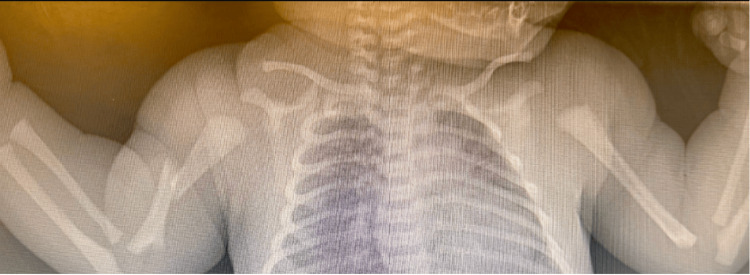
X-ray image after birth Acute appearing fracture of the right mid humeral diaphysis with mild angulation and 1.1 cm of a fracture fragment overlap. No other site of acute osseous trauma.

X-rays (Figure 1) were obtained and verified the diagnosis of right humeral fracture. As shown in Figure 1, there is a right mid-humeral diaphysis fracture with mild angulation and a 1.1 cm fracture overlap. There appears to be no other site of osseous trauma. The arm was immobilized by placing the infant in a long-sleeve shirt with the elbow bent to 90° flexion and pinned against the chest; no formal orthotic device was used. Liquid acetaminophen was prescribed as needed for pain. Spontaneous healing is expected. The baby was discharged home after three days.

**Figure 2:**
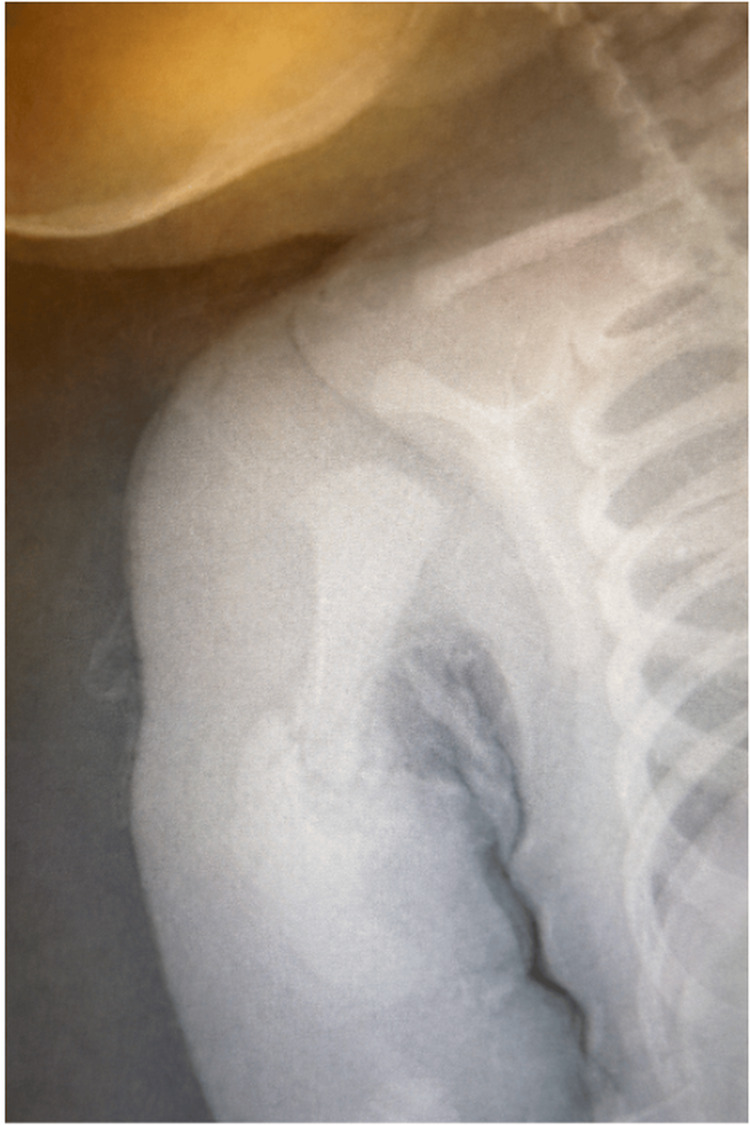
X-ray image after three weeks Healing fracture of the right humeral midshaft, with persistent angulation and proximal migration.

Upon three-week follow-up, X-ray (Figure 2) shows healing of the right humeral mid-shaft fracture with persistent angulation and proximal migration. At four-week follow-up, repeat neurological examination was normal with no evidence of late-onset weakness or brachial plexus injury.

## Discussion

Shoulder dystocia remains one of the most feared intrapartum emergencies due to its sudden onset, limited window for intervention, and potential for serious neonatal and maternal morbidity. Despite it being widely described as unpredictable and unpreventable, prompt recognition and systemic execution of evidence-based maneuvers are critical in minimizing adverse outcomes. This case illustrates both appropriate escalation of shoulder dystocia maneuvers and an uncommon neonatal complication, “humeral fracture”, following delivery of the posterior arm. Failure of the shoulder to deliver spontaneously places both the pregnant woman and fetus at high risk for permanent birth-related injury.

Brachial plexus injuries are among the most significant fetal complications associated with shoulder dystocia, occurring in approximately 4-16% of affected deliveries [7]. However, osseous injuries such as clavicular and humeral fractures may also occur, either as a direct result of impaction against the maternal pelvis secondary to the traction and rotational forces applied during obstetric maneuvers [8]. While clavicular fractures are more commonly reported, humeral fractures remain relatively uncommon, with an incidence ranging from 0.1% to 4.2% in cases of shoulder dystocia [8]. In this case, the absence of neurologic deficits, including a symmetric Moro reflex and preserved spontaneous movement of the affected extremity, helped distinguish the injury from a brachial plexus palsy. This distinction is clinically important, as brachial plexus injuries may have long-term implications, whereas neonatal long bone fractures generally have an excellent prognosis with conservative management. The presence of crepitus, localized swelling, and irritability with movement should raise clinical suspicion for fracture and prompt radiographic evaluation. Several other cases similarly described a mid-shaft humeral fracture following posterior arm delivery in the setting of shoulder dystocia, noting an equivalent absence of neurologic sequelae and full functional recovery with conservative immobilization, consistent with the outcome observed in our case [8,9].

The management of neonatal humeral fractures is typically non-operative. Immobilization using simple techniques such as pinning the arm to the chest or using a soft splint is usually sufficient, as neonates possess remarkable bone remodeling capabilities. Analgesia with acetaminophen is commonly used for comfort healing, which is rapid, often with radiographic evidence of callus formation within weeks, and long-term functional outcomes are overwhelmingly favorable [8]. Prior studies have demonstrated near-complete recovery without residual deformity in the majority of cases, even in the presence of initial angulation [10]. Clavicular fractures follow a similar healing trajectory to humeral fractures in neonates, with rapid callus formation, effective remodeling, and excellent long-term functional outcomes with conservative management. Notably, this case lacked quantitative data on exact traction forces applied or precise time intervals between individual maneuvers and delivery, which represent limitations in reproducibility and objective comparison with published series. Future case documentation should aim to capture these parameters, alongside patient-reported functional outcomes or validated neonatal motor scores at follow-up, to strengthen the evidence base for conservative management protocols.

This case also highlights the importance of balancing the urgency of delivery with the risk of iatrogenic injury. Delivery of the posterior arm, while effective in relieving shoulder dystocia, is associated with an increased risk of humerus fracture due to the mechanical forces applied during extraction. Nevertheless, this maneuver is often necessary and appropriate when less invasive techniques fail, as timely delivery remains critical to prevent hypoxic injury. In our case, five sequential maneuvers were performed within a 40-second interval (Table 1), reflecting appropriate algorithmic escalation under time pressure. Emerging evidence supports that adherence to a stepwise, algorithm-based approach reduces both neonatal morbidity and excessive traction forces during delivery [11]. Recent evidence from large-scale obstetric analysis further emphasizes that adherence to a structured, step-wise approach to shoulder dystocia management reduces excessive traction and improves neonatal outcomes, supporting the continued use of algorithm-based escalation in these emergencies [10].

## Conclusions

In conclusion, although neonatal humeral fracture is an uncommon complication of shoulder dystocia, it remains an important differential diagnosis in neonates presenting with upper extremity abnormalities after difficult delivery. Early identification through careful physical examination and appropriate imaging is essential to distinguish this injury from more serious neurologic conditions, such as brachial plexus injury. Prompt and appropriate conservative management, including immobilization and analgesia, typically results in rapid healing and excellent long-term functional outcomes due to the high remodeling capacity of neonatal bone. This case highlights that while established obstetric maneuvers are highly effective in resolving shoulder dystocia, they are not without risk and may contribute to rare iatrogenic injuries. Nevertheless, the benefits of timely intervention to prevent hypoxic complications far outweigh these risks when maneuvers are performed correctly.
